# Virus-informed targeting of HIV reservoirs: Rev-dependent HIV Immunotherapy (RHIT)

**DOI:** 10.1186/s12977-026-00682-9

**Published:** 2026-07-23

**Authors:** Yuntao Wu

**Affiliations:** Center for Infectious Disease Research, George Mason University, Manassas, VA 20110 USA

**Keywords:** HIV-1, HIV cure, HIV functional cure, Reservoir, Shock and kill, Rev, Tat, Rev-dependent vector, Immunotherapy

## Abstract

Persistence of viral reservoirs prevents a cure or functional cure for HIV. Efforts to eradicate viral reservoirs have been constrained by the difficulties of identifying and eliminating rare, heterogeneous viral reservoir cells in vivo. Single-modality interventions such as “shock and kill” have faced persistent challenges despite decades of effort, highlighting the need for complementary strategies that more precisely target infected cells. In this review, I summarize our 20 years of effort in developing an HIV Rev-dependent lentiviral vector for selective targeting of viral reservoirs. This lentiviral vector expresses therapeutic genes under the control of HIV Tat and Rev, conferring high specificity. In vitro, superinfection of HIV-infected cells with the Rev-dependent particle can lead to the identification and selective killing of infected cells. In vivo, injection of Rev-dependent lentiviral particles into SIV-infected rhesus macaques stimulates antiviral immunity, leading to reduction of viral reservoirs and inhibition of viral rebound. We designate this Rev vector-based therapeutic approach as Rev-dependent HIV Immunotherapy (RHIT). The RHIT approach integrates three complementary reservoir-targeting strategies: (1) RHIT-mediated killing of reservoir cells via Rev-dependent therapeutic gene expression; (2) RHIT-mediated inhibition of active HIV replication via defective interfering particles; and (3) RHIT-induced anti-HIV immunity via induction of neutralizing antibodies and cell-mediated immune responses. In this review, I also discuss limitations and challenges for the future development of RHIT.

## Introduction

More than 40 years into the global HIV/AIDS epidemic, significant progress has been made. However, 1.3 million new infections still occur annually, and approximately 600,000 people die from AIDS-related illnesses each year. HIV treatment remains a lifelong commitment. Additionally, people living with HIV are at increased risk for other diseases and conditions, such as active tuberculosis, central nervous system (CNS) and cardiovascular diseases, and cancers. To end AIDS, both a preventive vaccine and a cure (reservoir eradication) or a functional cure (long-term drug-free remission) are needed.

**Fig. 1:**
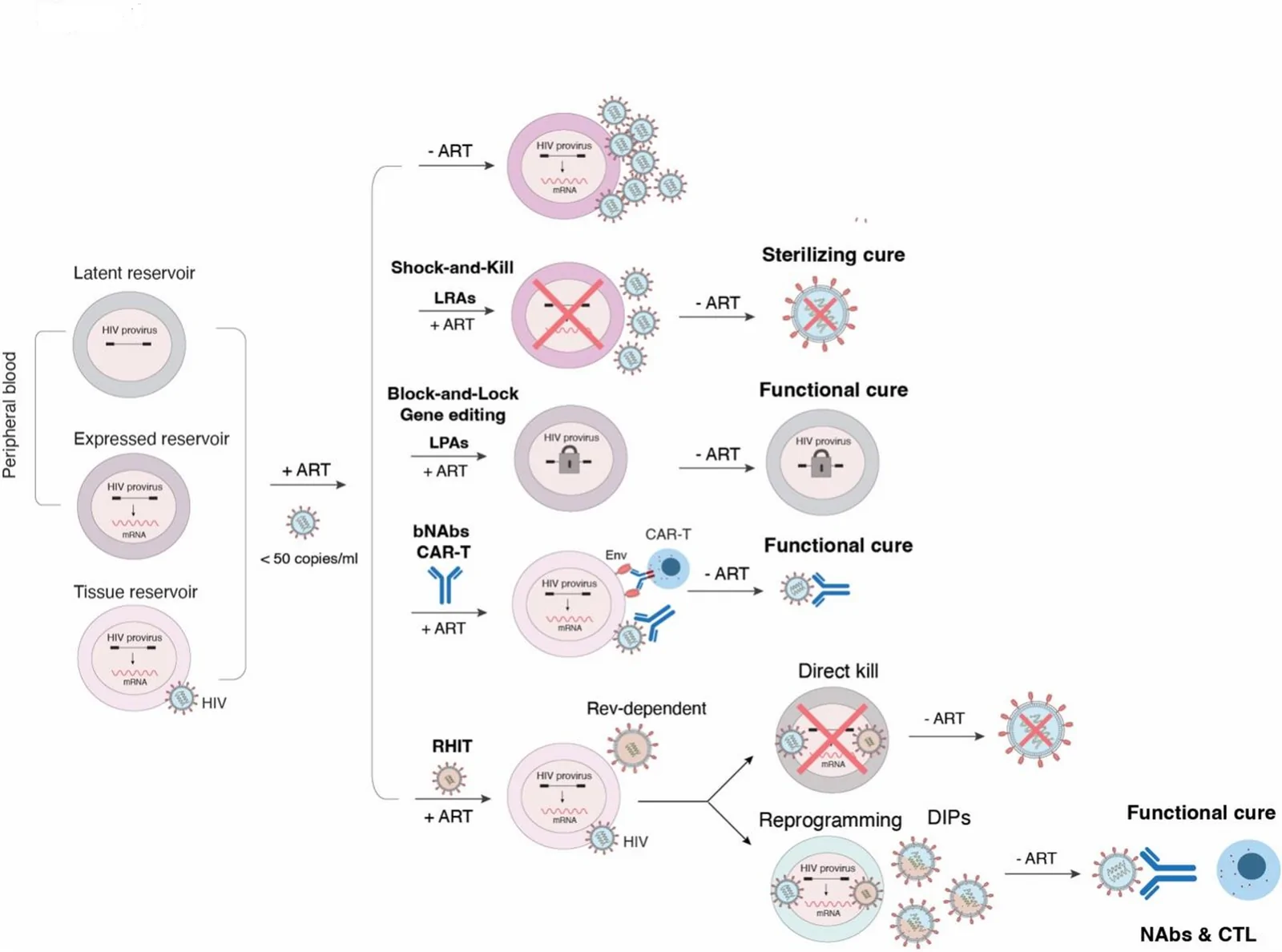
Illustration of RHIT and other strategies under development for an HIV cure. Peripheral HIV^+^ CD4 T cells harbor both latent (latent reservoir) and transcriptionally active (expressed reservoir) proviruses and represent the best-characterized viral reservoirs. HIV^+^ tissue reservoirs include tissue-resident macrophages, memory CD4 T cells, and follicular helper CD4 T cells. Although long-term antiretroviral therapy (ART) suppresses plasma viremia to below 20–50 copies/mL, it does not eliminate viral reservoirs, permitting spontaneous viral rebound upon ART interruption. The “shock-and-kill” strategy uses latency-reversing agents (LRAs) to induce proviral expression in the presence of ART, eliminating reactivated reservoir cells through viral cytopathic effects or immune-mediated killing by CD8 T cells and NK cells. The “block-and-lock” strategy employs latency-promoting agents (LPAs) to durably silence proviral transcription through epigenetic repression. Proviral silencing or disruption may also be achieved using gene-editing approaches such as CRISPR-Cas9. Immunotherapeutic strategies include broadly neutralizing antibodies (bNAbs), which block viral entry and replication to support long-term ART-free control, and genetically engineered chimeric antigen receptor (CAR)-T cells, which selectively target and eliminate HIV-infected cells. The RHIT strategy uses Rev-dependent lentiviral particles to superinfect HIV^+^ reservoir cells. Rev-dependent expression of therapeutic genes, such as HSV-1-tk, can directly kill reservoir cells. Additionally, the Rev-dependent vector can reprogram reservoir cells to produce defective interfering particles (DIPs), which suppress HIV replication while stimulating anti-HIV immunity, including neutralizing antibodies and cell-mediated responses

HIV rebound following antiretroviral therapy (ART) interruption remains the major barrier to achieving a cure or functional cure. Typically, HIV rebounds within 3–4 weeks after ART interruption [1, 2]. The exact sources of rebounding viruses have not been fully identified, although studies have demonstrated remarkable genetic heterogeneity among rebounding viruses, suggesting diverse reservoirs within the body [3]. Proliferation of reservoir cells has been suggested as a major reason for viral persistence and rebound after long-term ART [4–6]. Elimination or inactivation of these reservoirs is the primary goal for achieving a cure or functional cure. For more than a decade, HIV cure research has been dominated by the “shock and kill” paradigm, which seeks to pharmacologically reverse latency and rely on immune-mediated clearance. However, increasing evidence indicates that latency reversal is incomplete and heterogeneous [7], particularly within tissue reservoirs, and that reactivated cells frequently evade elimination due to insufficient antigen expression and dysfunctional HIV-specific immunity. To date, these promising approaches have yet to achieve durable ART-free remission in humans. In parallel, immune-based strategies, including broadly neutralizing antibodies, have demonstrated that long-term viral control may be achievable without a sterilizing cure. These findings highlight the need to reconsider therapeutic paradigms that are explicitly informed by the biology of viral persistence. In this review, I discuss virus-informed approaches, where therapeutic activity is coupled directly to viral gene expression, representing a complementary and underexplored strategy for targeting HIV reservoirs. Specifically, this review is centered on the Rev-dependent vector platform, RHIT (Rev-dependent HIV immunotherapy), that has been in development for the past 20 years [8–15]. By restricting therapeutic activity to cells actively expressing HIV Rev, RHIT selectively targets infected cells while sparing uninfected bystanders. This review also describes how RHIT integrates multiple mechanisms, including direct elimination of infected cells, interference with viral replication, and stimulation of antiviral immune responses. Recent nonhuman primate studies provide the first in vivo proof of concept that such Rev-dependent approaches can reduce viral rebound following ART interruption. Beyond discussing a single cure platform, this review situates virus-informed targeting strategy within the broader landscape of HIV cure research, drawing comparisons with latency reversal, immune modulation, and combination approaches (Fig. 1). However, the scope of this review is limited in its coverage of these more extensively developed strategies, as its primary focus is the recent development of the Rev-dependent vector [8]. For in-depth comprehensive reviews of widely studied HIV cure strategies, including “shock-and-kill” and “lock-and-block”, readers are referred to prior reviews by Marsden and Zack [16], Deeks [17], Kim et al. [18], Flemming [19], Abner and Jordan [20], and Ahlenstiel et al. [21].

## HIV reservoirs

In patients receiving ART, several cell types have been identified to harbor intact proviruses or replication-competent HIV, most notably the peripheral blood CD4 T cells including memory, naïve, and circulating T follicular helper CD4 T subsets [4, 22–27], which are major targets of HIV [28, 29]. In addition, myeloid cells such as macrophages/monocytes, the other targets of HIV [30–32], have also been found to carry intact proviruses [33–37]. HIV DNA and proviruses have been detected in multiple tissues [38–40], including lymph nodes, brain, tonsillar tissue, intestinal tissue, and male and female reproductive tissues such as testes and cervix [41–50]. Importantly, viral reservoirs located within tissue sanctuaries are particularly challenging because they are difficult for antiretroviral drugs to penetrate and may be less responsive to certain therapeutic interventions such as treatment with HDAC inhibitors [47, 51].

Among HIV reservoirs, peripheral blood resting CD4 T cells are the most extensively studied. This reservoir was suggested to exist predominantly in a latent form, which may be established either through direct infection of resting T cells [52–54] or through the transition of actively infected T cells back to a resting state [55]. In vitro, direct infection of resting T cells is inefficient and requires overcoming multiple cellular barriers, including low intracellular metabolic activity and dNTP levels [56], the presence of restriction factors such as SAMHD1 [57–59], and limited chemokine-induced cortical actin dynamics required for viral entry and nuclear trafficking [53, 54]. Maintenance of proviral latency in resting T cells also involves multiple mechanisms, including the formation of restrictive chromatin structures at the LTR [60], the absence of critical transcription factors such as NF-κB and NFAT [61, 62], recruitment of histone deacetylase 1 (HDAC1) to the LTR by host factors such as YY1 and LSF to suppress transcription [63], and sequestration of P-TEFb within the inactive 7SK RNP (7SK small nuclear ribonucleoprotein) complex [64]. In addition, the site of proviral integration can influence the establishment and maintenance of latency. For example, direct sequencing of integration sites has shown that latent proviruses are frequently integrated within or near alphoid repeat elements in heterochromatin, suggesting that HIV can establish latency following integration in repressive chromatin environments [65]. Proviral latency may also arise through transcriptional interference from host gene transcription that disrupts activity of a nearby proviral promoter [66].

Immune selection also plays an important role in shaping the persistence of HIV reservoirs; infected cells that express higher levels of viral antigen are preferentially eliminated by cytotoxic CD8 T cells and NK cells, while cells with minimal antigen expression are more likely to persist. This process may progressively enrich for proviruses that are transcriptionally silent or deeply latent, often associated with repressive chromatin environments that limit viral gene expression and immune recognition [67, 68]. Over time, intact proviruses become increasingly concentrated in long-lived memory CD4 T cell subsets and clonal expansions, reflecting both homeostatic proliferation and antigen-driven expansion of infected cells [69]. Such dynamics result in a reservoir that is highly enriched for poorly inducible proviruses that escape immune clearance under antiretroviral therapy [70]. Overall, immune pressure may act progressively to remove active or visible infected cells while favoring persistence of deeply latent, immune-evasive clones that are characterized with MHC class I downregulation or cell survival receptor upregulation [71–73].

## Overview of strategies to target HIV reservoirs

Although ART effectively suppresses plasma viremia, it is not effective in reducing viral reservoirs [43, 74, 75]. A fundamental question is why HIV rebound always occurs spontaneously in patients within weeks after ART termination, without reliance on external stimuli (e.g., co-infection or stimulation with drugs such as HDAC inhibitors). A reasonable explanation is that there are proliferation-driven stable reservoir cells that are active in synthesizing some low levels of viruses or viral proteins [1, 76–80]. Maldarelli et al. have demonstrated that these long-lived virus-producing reservoir cells are not affected by ART [80], but can seed a spread infection of new target cells upon ART termination [79, 81]. Targeting these reservoirs likely holds the key for viral eradication or treatment-free remission [76].

The most studied viral reservoirs, peripheral HIV^+^ CD4 T cells, contain both transcriptionally active (expressed reservoir) and latent (latent reservoir) HIV [76, 82]. A unique feature of the peripheral latent reservoir is that resting T cells negligibly express viral genes, requiring T cell activation or immune stimulation to initiate viral replication. The persistence of latency is largely governed by the HIV proviral LTR’s dependence on cellular transcription factors (such as NF-κB and SP1), regulatory proteins (such as P-TEFb), and the surrounding chromatin environment. In the absence of transcriptional stimulation of the particular chromatin region where the provirus resides, HIV may remain latent in resting T cells for decades, with estimates suggesting persistence for up to 60 years [74]. To address latency, the “shock-and-kill” strategy has been proposed and is being actively pursued (Fig. 1). This approach aims to activate latent reservoirs, triggering viral gene expression and enabling the immune system or viral cytopathic effects to eliminate infected cells. The search for effective LRAs has led to the development and testing of various compounds, including HDAC inhibitors and PKC activators [83–91]. While these agents can induce HIV gene expression in some latent cells, they do not consistently result in the death of infected cells [87–91]. For instance, a clinical study with the HDAC inhibitor vorinostat (VOR) demonstrated that a single dose of VOR induced an increase in HIV RNA expression in resting CD4 T cells but did not substantially reduce HIV^+^ CD4 T cells [91].

The lack of demonstrated clinical efficacy may be attributed to several factors. First, current LRAs do not sufficiently reactivate all latent reservoirs because of the extensive heterogeneity of latently infected cells. Second, even when latent cells are “shocked,” the host immune response, particularly CD8 T cells, is often too exhausted or functionally impaired to eliminate them effectively. In addition, recent findings indicate that HDAC inhibitors fail to induce HIV expression in multiple tissues, including tonsillar, intestinal, and cervical tissues [40]. Moreover, given the broad anatomical distribution and cellular diversity of HIV reservoirs, a persistent challenge for the “shock-and-kill” strategy is achieving effective activation of latent reservoirs without triggering immunopathology or excessive bystander immune activation.

In addition to HDAC inhibitors, several other classes of LRAs have been investigated. Protein kinase C (PKC) agonists, including bryostatin-1, prostratin, and ingenol derivatives, can activate NF-κB signaling and induce potent HIV transcription [91–93]. Bromodomain and extra-terminal (BET) inhibitors such as JQ1 and I-BET151 have also been shown to reverse latency by releasing BRD4-mediated repression and promoting P-TEFb-dependent transcriptional elongation [94–96]. More recently, SMAC mimetics, including AZD5582 and birinapant, have emerged as promising agents that activate the non-canonical NF-κB pathway and induce HIV expression in vitro and in animal models [97, 98]. Toll-like receptor (TLR) agonists, particularly TLR7 and TLR9 agonists such as vesatolimod (GS-9620) and lefitolimod (MGN1703), have also been explored for their dual ability to promote latency reversal and enhance antiviral immune responses [99, 100]. In addition, immune-modulatory agents such as the IL-15 superagonist N-803 have shown potential to couple reservoir activation with enhancement of NK and CD8 T cell-mediated clearance [97, 101]. Other emerging approaches include histone methyltransferase inhibitors, DNA methyltransferase inhibitors, and other epigenetic modulators targeting complementary mechanisms of latency maintenance [102, 103]. Despite these advances, no single LRA has yet achieved robust and consistent reduction of the latent reservoir in clinical studies, and current efforts increasingly focus on combination regimens designed to enhance both the “shock” and “kill” components of this strategy.

As an alternative to the “shock-and-kill” approach, the “block-and-lock” strategy has been proposed to achieve a functional cure for HIV by permanently silencing latent viral reservoirs [21, 104] (Fig. 1). This method aims to lock HIV proviruses into a lasting epigenetically repressed state, preventing reactivation and viral rebound [21, 104]. One example is the Tat inhibitor didehydro-cortistatin A (dCA), which has been investigated for its ability to silence HIV transcription [105]. In a BLT mouse model, dCA treatment delayed viral rebound for up to 19 days following ART cessation [106]. Other compounds, such as the HSP90 inhibitors tanespimycin and AUY922, have also demonstrated efficacy in delaying viral rebound, with effects lasting up to 11 weeks in HIV-infected humanized mice [107].

In addition, CRISPR-Cas9-mediated gene editing has been used to directly disrupt HIV proviruses in infected cells [108–114]. Animal model studies have demonstrated in vivo safety and efficacy of excising HIV-1/SIV proviruses [115–117]. Nevertheless, a recent clinical study, EBT-101, demonstrated that while the therapy is well tolerated, it does not prevent HIV-1 rebound in patients (Clinicaltrials.gov ID: NCT05144386). As a promising strategy, several major limitations have hindered clinical translation of CRISPR-Cas9 for HIV cure. First, the extensive genetic diversity of HIV and the potential for CRISPR-induced mutations at target sites can lead to viral escape, reducing long-term efficacy, particularly when single guide RNAs are used [118]. Second, efficient delivery of CRISPR components to the diverse and widely distributed latent reservoirs, including resting CD4 T cells and tissue reservoirs, remains a major challenge. Third, incomplete excision of proviral DNA and off-target genome editing raise concerns about residual replication-competent virus, genotoxicity, and safety [112, 115]. In addition, latent proviruses embedded in restrictive chromatin may be less accessible to Cas9-mediated editing, limiting efficacy in deeply latent reservoirs [68, 119]. Finally, because CRISPR alone is unlikely to eliminate all rebound-competent reservoirs, it is increasingly viewed as a component of combination cure strategies rather than a standalone intervention [108].

Broadly neutralizing antibody (bNAb)-based immunotherapies are also being actively developed with the goal of achieving long-term HIV remission in the absence of ART [120–127]. bNAbs function by neutralizing a broad spectrum of HIV variants through binding to highly conserved regions of the viral envelope (Env). These regions include five primary sites: the CD4 binding site, the V1/V2 trimer apex, the gp120-gp41 junction, the high-mannose patch, and the membrane-proximal external region [128]. bNAbs offer several advantages, such as prolonged viral load control, the potential for month-long dosing intervals rather than daily pills, and the ability to stimulate broader immune responses, including the engagement of natural killer (NK) cells and stimulation of CD8 T cells. Despite these benefits, clinical use of bNAbs is challenged by issues such as pre-existing viral resistance, variations in antibody durability, and limited efficacy against latent HIV reservoirs. Future applications of bNAbs will likely require combination strategies, such as pairing with vaccines or viral vector-based approaches to induce endogenous bNAbs and CD8 T cells [121, 129, 130, 130].

In addition to bNAbs, chimeric antigen receptor (CAR)-T cell therapy is being explored as a promising immunotherapeutic strategy for eliminating HIV reservoirs [131–133]. In this approach, autologous T cells are genetically engineered to express CARs that recognize HIV envelope proteins on infected cells, enabling antigen-specific killing. In a clinical study, infusion of VRC01-derived bNAb CAR-T cells was shown to be safe. Although viral rebound occurred in all six participants following ART interruption, cell-associated viral RNA and intact proviruses decreased significantly after CAR-T cell treatment, suggesting partial reservoir reduction and antiviral activity [134]. More recent CAR-T designs have incorporated co-stimulatory domains, such as CD28 and 4-1BB, to enhance expansion, persistence, and effector function [135], as well as resistance mechanisms such as CCR5 disruption to protect CAR-T cells from HIV infection and improve potency and durability [136, 137]. Additional strategies, including multi-specific or duoCAR-T constructs designed to target conserved Env epitopes and reduce viral escape, are also under investigation [136]. Despite this promise, important challenges remain, including viral escape, limited trafficking to anatomical reservoirs, T cell exhaustion, and safety considerations.

## Targeting HIV reservoirs with Rev-dependent HIV immunotherapy (RHIT)

Over the past two decades, we and others have been developing an HIV Rev-dependent lentiviral vector designed for selective targeting of viral reservoirs [8–14] (Fig. 1). This vector drives therapeutic gene expression under the control of the viral regulatory proteins Tat (trans-activator of transcription) and Rev (regulator of virion protein expression), thereby conferring high specificity, as only HIV-infected cells express both factors. Tat is among the earliest viral proteins produced following infection and serves as a central activator of HIV transcription, whereas Rev governs the export and expression of late viral transcripts. The molecular functions and regulation of Tat and Rev have been extensively characterized in past decades. For comprehensive reviews, readers are referred to Jones and Peterlin [138], Karn [139], Rice [140], Pollard and Malim [141], Rausch et al. [142], and Truman et al. [143]. A brief overview of Tat and Rev function is provided below to facilitate understanding of the Rev-dependent vector design.

### Tat and Rev function as key regulators of HIV transcription

**Fig. 2:**
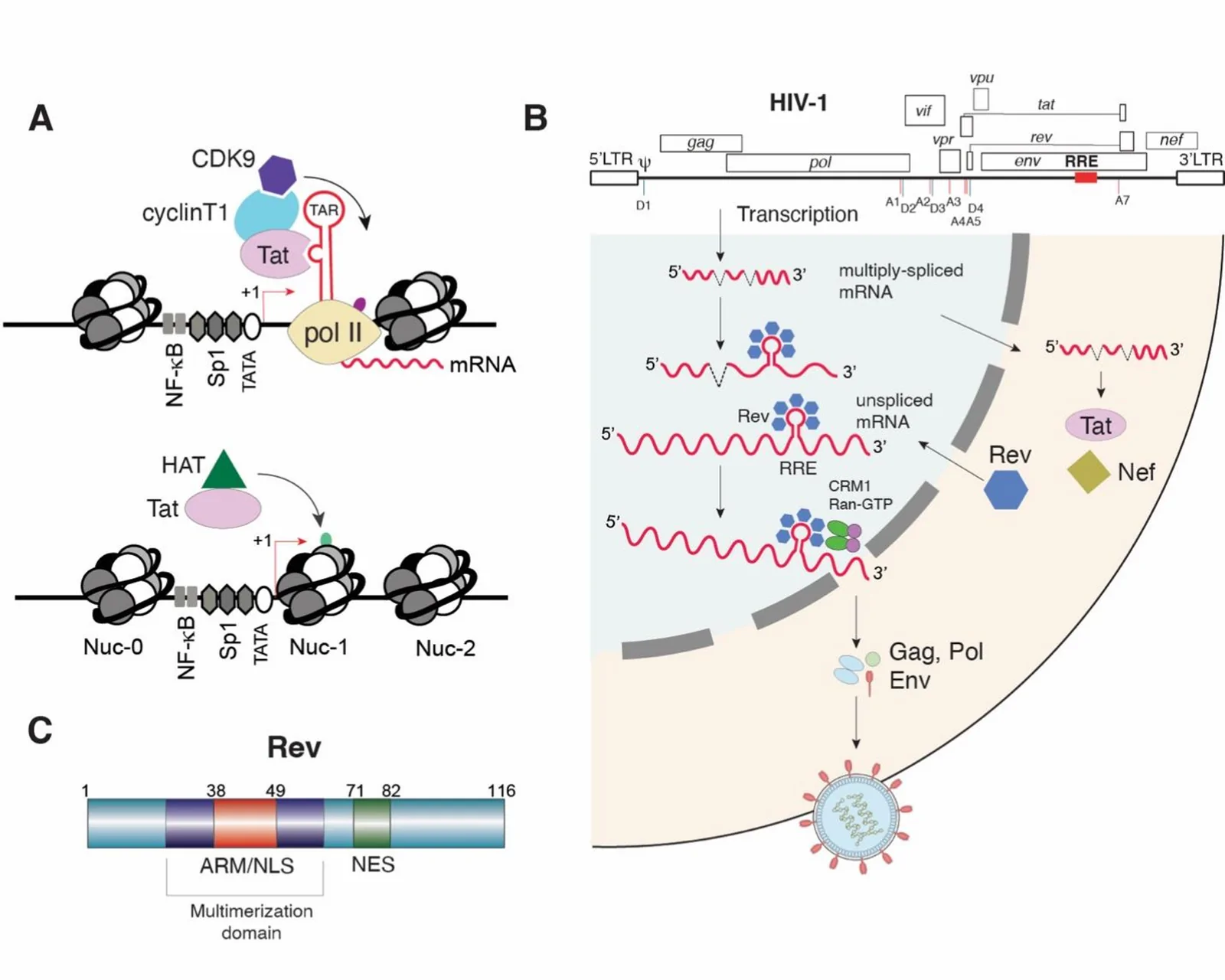
Regulation of HIV transcription by Tat and Rev. **A** Regulation of early HIV transcription by Tat. During early HIV gene expression, basal transcription from the HIV long terminal repeat (LTR) generates short, inefficient transcripts due to promoter-proximal pausing of RNA polymerase II. The HIV Tat protein binds the TAR RNA element at the 5′ end of nascent transcripts and recruits the cellular P-TEFb complex (Cyclin T1/CDK9), promoting phosphorylation of RNA polymerase II and pausing factors to stimulate transcriptional elongation. This relieves promoter-proximal pausing, increases full-length viral transcript production, and amplifies HIV gene expression through a positive feedback loop (top). In addition, Tat stimulates HIV transcription through recruitment of histone acetyltransferases (HATs) to remodel nucleosomally assembled LTR chromatin, facilitating assembly of general transcription factors and transcription initiation (bottom). **B** Regulation of HIV late gene expression by Rev-mediated nuclear export of singly spliced and unspliced viral RNAs. Following transcription from the HIV provirus, viral RNAs are initially retained in the nucleus unless fully spliced. Multiply spliced early mRNAs direct synthesis of Tat, Rev, and Nef. Rev binds the Rev response element (RRE) within unspliced and singly spliced viral RNAs, multimerizes, and recruits the cellular export receptor CRM1/exportin 1 in a Ran-GTP-dependent manner to mediate nuclear export. This process enables cytoplasmic transport of incompletely spliced viral RNAs for translation of structural and enzymatic proteins (e.g., Gag, Pol, and Env) and for packaging of genomic RNA into virions. **C** Domain organization of HIV-1 Rev. Rev is a 13-kDa protein composed of 116 amino acids and contains two key functional domains involved in nuclear trafficking. The N-terminal arginine-rich motif (ARM; amino acids 38–49) mediates RNA binding and Rev multimerization and contains a nuclear localization signal (NLS) for nuclear import. The C-terminal leucine-rich nuclear export signal (NES; amino acids 71–82) mediates CRM1-dependent nuclear export

Tat functions as a transcription elongation factor by recruiting P-TEFb (positive transcription elongation factor b) to overcome promoter-proximal pausing (Fig. 2A). Tat regulates HIV transcription by two major mechanisms. Firstly, Tat promotes the processivity of HIV transcription by recruiting P-TEFb, a protein complex mainly composed of cyclin T1 and CDK9, which hyperphosphorylates the carboxyl-terminal domain (CTD) of the large subunit of RNA polymerase II; this leads to promoter-proximal pause release and processive elongation [144–148]. A unique feature of Tat is that it interacts with RNA rather than with DNA [149]. Tat directly binds to TAR (Trans-activation Response element, nt + 1 to + 60), a specific 60-residue stem-loop structure on the RNA leader sequence which serves as the docking site for the Tat and P-TEFb complex [139, 150, 151]. Secondly, Tat modulates HIV LTR chromatin, both prior to HIV genome integration and post-integration [65, 152–156]. Tat has been shown to interact with the p300/CBP and P/CAF histone acetyltransferases (HAT) [153, 157, 158], and other HATs such as Tip60 [159] and hGCN5 [160]. Tat-mediated remodeling of nucleosomes can relieve transcriptional blockage imposed by chromatin. In addition, Tat has also been suggested to directly interact with some transcription factors such as Sp1 [156, 161] and TBP [162] to promote transcription.

HIV transcription from provirus is orchestrated by sequential alternative-splicing. A single, full-length mRNA is transcribed and fully spliced early into the multiply-spliced mRNAs that express early proteins including Tat and the second regulatory protein, Rev (regulator of expression of virion proteins). Rev functions to mediate the cytoplasm localization of the unspliced and singly-spliced HIV transcripts, such as the env transcript, for protein synthesis [163] (Fig. 2B). With the increase of Rev in infected cells, both the singly-spliced and unspliced mRNAs accumulate and are exported to the cytoplasm for translation into multiple structural proteins including Gag, Pol, Env, Vif, Vpu, and Vpr. Rev regulates HIV late gene expression [164, 165] mainly through mediating the nuclear export of the singly-spliced and unspliced viral mRNAs [163, 166]. While the multiply-spiced mRNAs are exported by the default cellular NXF1-mediated mRNA export pathway [167–169], the singly-spliced and unspliced mRNAs are retained in the nucleus as intron-containing mRNAs and require Rev for their nuclear export [143, 166, 170].

Structurally, Rev contains several functional domains including the N-terminal domain (NTD) and the C-terminal domain (CTD) (Fig. 2C). NTD contains two oligomerization motifs, an arginine-rich RNA-binding motif (ARM), and a nuclear/nucleolar localization signal (NLS), while CTD contains a nuclear export signal (NES) [143]. Rev directly binds to an RNA structure, the Rev Responsive Element (RRE), that is present only in the singly-spliced and unspliced viral mRNAs within the Env gene region [166]. RRE consists of approximately 350 nucleotides, is highly structured, and is predicated to fold into 4 or 5 stem-loops (SL) (I, II, III, IV, V). Rev oligomers bind sequentially to RRE stem-loop regions such as stem-loop II via its ARM motif and the oligomerization domains [171–178]. The Rev oligomers have a 500-fold higher affinity for RRE than a single Rev binding to RRE [172, 174–176]. This highly orchestrated Rev oligomeric interaction with RRE is required for viral mRNA nuclear export, allowing the recruitment of cellular exportin CRM1 (Chromosomal Region Maintenance Gene 1) and RanGTP to the Rev-mRNA complex through binding to the Rev NES domain [175, 179, 180]. The formed Rev-mRNA-CRM1-RanGTP complex uses the hydrophobic channel of the nuclear pore for mRNA nuclear export [181–183]. In the cytoplasm, Rev dissociates from mRNA through RanGTP hydrolysis, and recycles back into the nucleus through cellular importin β, transportin, importins 5 and 7, and ribosomal assembly factor B23 [184–187].

Beyond its canonical role in mediating nuclear export of viral mRNA, Rev has been implicated in the regulation of HIV RNA processing at multiple levels. Rev appears to modulate mRNA splicing, as the presence of 5’ and 3’ splice sites influences its activity [188, 189]. Consistent with this, a peptide derived from the Rev RNA-binding domain selectively inhibits in vitro splicing of RRE-containing pre-mRNA [190], blocking the entry of U4, U5, and U6 small nuclear RNAs into the spliceosome and leading to accumulation of a 45–50 S splicing-deficient complex [191]. Mechanistically, Rev-mediated splicing inhibition may involve recruitment of the essential splicing factor SF2/ASF to the Rev/RRE complex; SF2/ASF binds a defined RRE subregion in a Rev-dependent manner [189, 192]. In addition, Rev has also been proposed to harbor a UHM ligand motif within its arginine-rich motif, enabling interactions with UHM-containing splicing factors such as U2AF65 and SPF45, and potentially influencing HIV transcript splicing [193].

Rev has also been suggested to regulate HIV late gene expression at the level of translation by promoting HIV mRNA polysomal association for protein translation [194–197]. Notably, in a nuclear expression system, Rev increased env mRNA export by approximately 1.6 fold but enhanced translation by more than 100 fold [196]. Beyond the RRE, Rev was also found to bind to a purine loop in the packaging signal SL1 region of the 5’ UTR of HIV-1 RNA [198], an interaction implicated in regulating viral genomic RNA dimerization and packaging [199–201]. Consistently, Rev-RRE interaction has also been shown to affect viral genomic packaging [201–204]. In some context, Rev elevates cytoplasmic levels of unspliced HIV-1 RNAs 3 to 12 fold, yet stimulates genome encapsidation by over 1,000 fold [205]. Despite these observations, the precise molecular mechanisms and their relationship with Rev’s canonical function remain to be fully defined.

### Rev-dependent lentiviral vector and its specificity in mediating gene expression in HIV^+^ cells

**Fig. 3:**
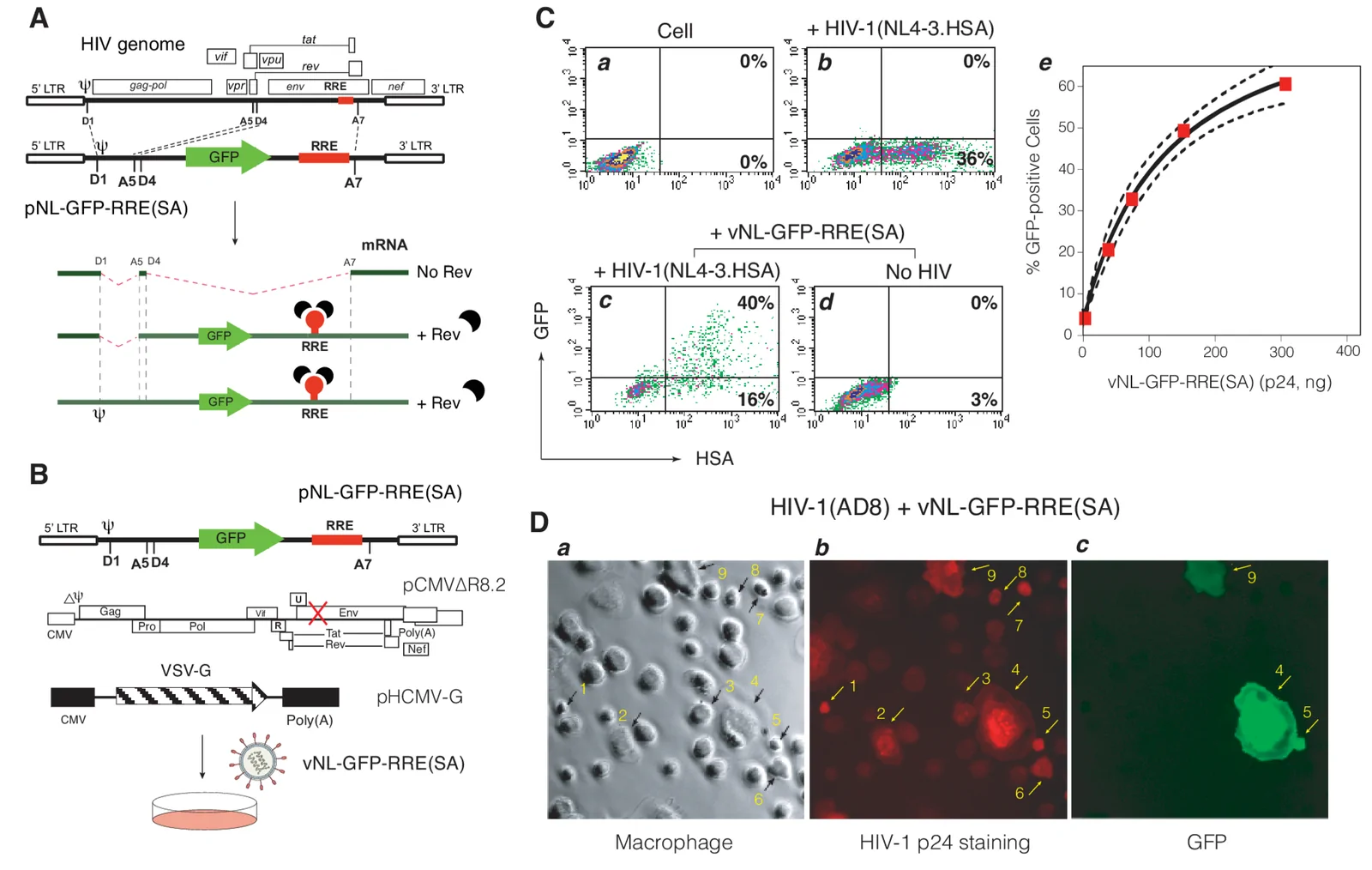
Illustration of the Rev-dependent vector pNL-GFP-RRE(SA) and its capacity to selectively target HIV-infected cells. **A** The vector transcribes mRNAs from the LTR promoter. In the absence of HIV Rev, all transcripts are spliced. In the presence of HIV and Rev, Rev binds to RRE, preventing splicing, and mediating the nuclear export and translation of the unspliced and singly-spliced GFP-containing mRNAs. **B** Assembly of the Rev-dependent vector particles, which is achieved by co-transfection of HEK293T cells with the Rev-dependent vector, and vectors expressing the HIV structural protein (pCMV∆R8.2) and the VSV-G glycoprotein (pHCMV-G). **C** High specificity of the Rev-dependent vector. CEM-SS T cells were not infected (**a**) or singly infected with HIV-1(NL4-3.HSA), a recombinant HIV expressing the HSA (mouse heat-stable antigen) surface antigen (**b**). Following HIV infection, cells were also superinfected with vNL-GFP-RRE-SA at 24 h (**c**). For comparison, cells were also singly infected with either vNL-GFP-RRE-SA (**d**). Both HSA and GFP expression were analyzed at 72 h. In **e**, HIV-infected cells (HSA^+^) were superinfected with varied dosages of vNL-GFP-RRE-SA (x-axis). Flow cytometry yielded percent HIV-positive cells that became GFP-positive after 3 days (y-axis). Non-linear curve fitting (rectangular hyperbola) yielded R2 = 99.4; maximum GFP-positive = 86.1 ± 6.3% (best-fit values ± standard error); KD = 128.1 ± 21.5 ng p24. Dashed lines are 95% confidence bands. **D** Selective expression of the Rev-dependent GFP reporter only in HIV-infected macrophages. Blood monocyte-derived macrophages were infected with HIV(AD8), followed by superinfection with vNL-GFP-RRE-SA. **D**, **a** Light microscopy of infected macrophages. **D**,** b** The same cells were stained for HIV-1 p24 with a PE-labeled antibody (red fluorescent). **D**,** c** The same field is also observed for GFP. Adapted with permission from BioMed Central Ltd. *Retrovirology* 4, 12 (2007) [8]

Regardless of the mechanistic complexity, the well-established viral requirement for Rev has led us to use the Rev-RRE interaction as the regulator to achieve selective expression of therapeutic genes only in HIV-infected cells. For this purpose, we constructed the HIV Rev-dependent vector (available from the NIH AIDS Reagent Program, Cat# 11466), in which a therapeutic or reporter gene is expressed from the HIV-1 L (long terminal repeat) promoter, and regulated by Rev by placing the gene before RRE and between the multiple authentic HIV splicing sites (D1, A5, D4, A7) (Fig. 3A). This arrangement regulates the therapeutic or reporter gene as a late gene, similar to Gag or Env, and renders its expression fully dependent on Tat and Rev. The use of authentic HIV splicing donors and acceptors is to minimize potential impacts of the unnatural splicing site on Rev function [188, 189]. The Rev-dependent vector also contains the viral genomic RNA packaging signal (ψ). Thus, when cotransfected with HIV structural protein expression vectors, its transcript will be packaged into virion particles as the genomic RNA [8] (Fig. 3B). The assembled particles can be used to superinfect HIV^+^ cells.

The high specificity of the Rev-dependent lentiviral vector in the selective expression of reporter genes has been demonstrated in HIV-infected T cells and macrophages [8, 9] (Fig. 3C and D). When the Rev-dependent GFP lentiviral particle was used to superinfect HIV-infected CEM-SS T cells, it did not generate GFP-positive cells without HIV-1 co-infection, even with a vector multiplicity of infection (MOI) as high as 10, demonstrating the strict requirement for HIV to trigger reporter gene expression. In addition, the percent of HIV-infected cells that expressed GFP increased proportionally with increasing reporter viral particle input. The curve fit is a rectangular hyperbola, defining this as a saturable process, and predicts that 80–90% of the HIV-infected cells can be targeted by the Rev-dependent GFP reporter virus (Fig. 3C). In the superinfection of HIV-infected macrophages, only HIV-positive cells (p24^+^) displayed GFP, and none of the HIV-negative cells displayed GFP, when the Rev-dependent GFP reporter virus was used at a low dosage (MOI = 0.2–0.5), demonstrating that a single infecting GFP-indicator viral particle is capable of detecting the presence of HIV (Fig. 3D) [8].

Gene expression from the Rev-dependent particle has been shown to be regulated at the level of mRNA splicing [8, 190, 191]. When delivered into uninfected cells, the vector transcribes low levels of mRNAs from the LTR promoter. However, in the absence of HIV, these mRNAs are spliced into small, non-coding mRNAs. On the other hand, when the vector is delivered into HIV-infected cells, in the presence of Rev, splicing is inhibited, and the unspliced, partially-spliced RRE-containing mRNAs accumulate and are detected [8].

The Rev-dependent vector has also been used in multiple laboratories to generate HIV Rev-dependent reporter cell lines [9, 206–211]. These cells have been used for studying HIV infection, viral tropism, Rev inhibitor, viral drug resistance, neutralizing antibodies, restriction factors, and HIV cell-cell transmission [206, 211–217]. Consistent with the high specificity observed in the superinfection studies [8], the Rev-dependent reporter cells carrying integrated copies of the Rev-dependent vector are highly responsive to HIV infection, but minimally respond to non-viral stimuli such as mitogens and cytokines [9, 211, 213, 217].

### RHIT-mediated killing of HIV reservoir cells via Rev-dependent therapeutic gene expression

We have tested the capacity of Rev-dependent particles for killing HIV-infected cells using a series of therapeutic genes with different mechanisms of action and safety profiles. These include anthrolysin O (AlnO) from *Bacillus anthracis*, diphtheria toxin A chain (DT-A) from *Corynebacterium diphtheriae*, the endogenous pro-apoptotic human gene TRAF6 (tumor necrosis factor receptor-associated factor 6), and the herpes simplex virus type 1 thymidine kinase (tk) gene (HSV-1-tk) [12,218, 219]. Our in vitro studies demonstrated that Rev-dependent particles carrying these genes can selectively kill HIV-infected T cells and macrophages while leaving uninfected cells unharmed [10, 11]. The Rev-dependent vector, pNL-GFP-RRE-SA, has also been used by the Joshi laboratory to express a HSV-1-tk mutant, TK-SR39, and delivered into Jurkat T cells, which led to the suppression of HIV-1 replication and virus-induced cytopathic effects following ganciclovir (GCV) treatment [13]. In addition, the Rev-dependent TK-SR39 vector has also been combined with a non-integrating lentiviral vector to express a CCR5gRNA-CRISPR/Cas9 cassette and HIV Tat protein. The two-vector system has been delivered into TZM cells and generate a cell line that is resistant to CCR5-tropic viruses while permitting the infection of CXCR4-tropic viruses which were suppressed via the treatment with GCV [14]. These in vitro experiments demonstrate the potential of the Rev-dependent vector in selective killing of HIV^+^ cells via Rev-mediated expression of cytotoxic genes.

Recently, we conducted a proof-of-concept study in rhesus macaques using SIV (simian immunodeficiency virus) Rev-dependent lentiviral particles carrying the HSV-1-tk gene. The particles were injected into SIV-infected animals to target viral reservoirs and inhibit viral rebound after antiretroviral therapy (ART) termination [12].

**Fig. 4:**
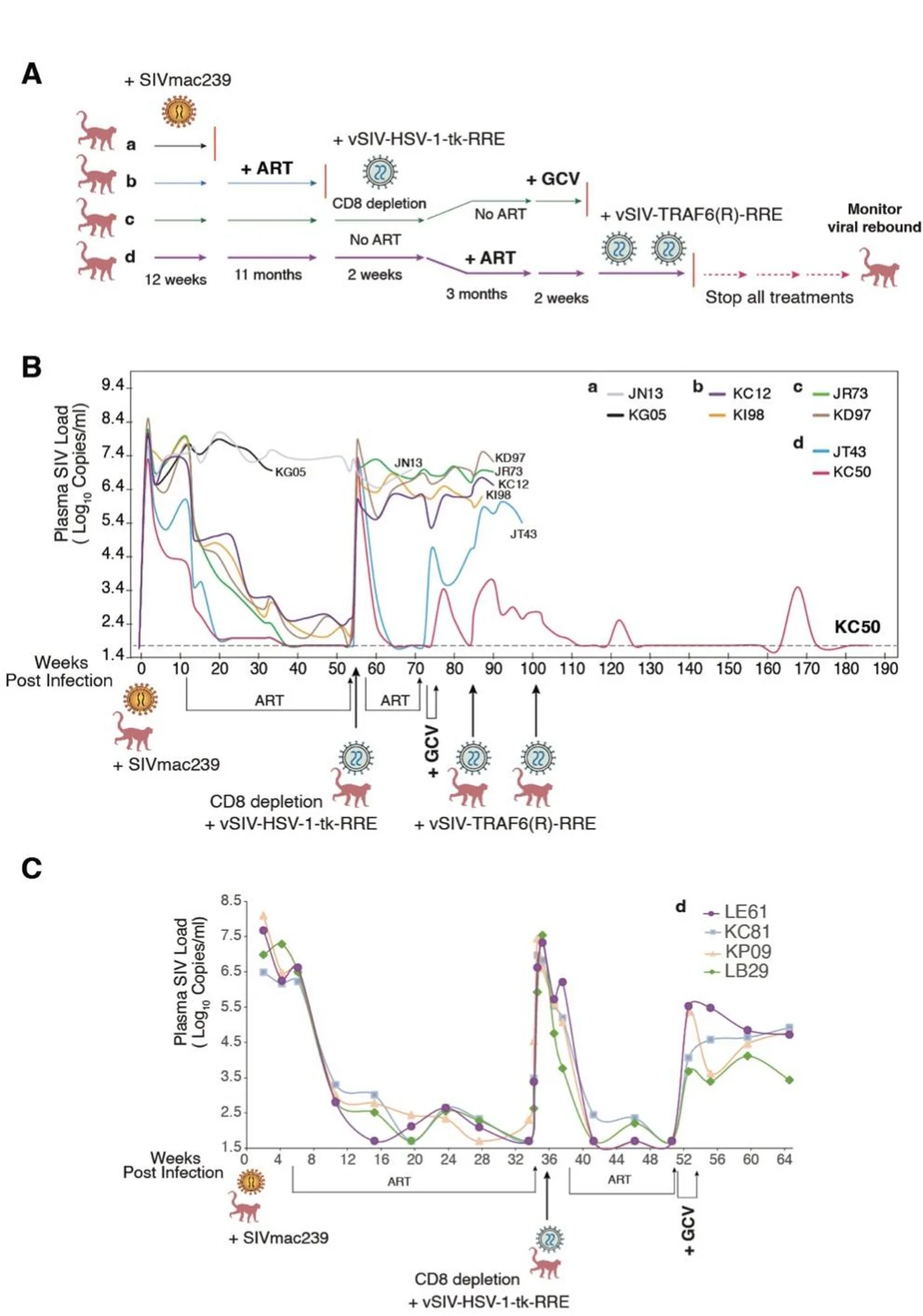
Proof-of-concept in vivo testing of the SIV Rev-dependent vectors in SIVmac239-infected Indian rhesus macaques. **A** Schematic of the different treatments and vector injection designs for study group **a** to **d**. (*n* = 2 for each group). For Group **a**, monkeys were infected with SIVmac239, but received no ART. For Group **b**, animals were similarly infected with SIV but were treated with ART for 11 months. ART was then terminated to allow viral rebound to occur. For Group **c**, animals were infected and treated with ART for 11 months. ART was then stopped, and animals were injected with vSIV-HSV-1-tk-RRE particles. ART was not resumed in the animals after vSIV-HSV-1-tk-RRE injection. This was to permit vector amplification and mobilization with active SIV replication. Group **d** animals were treated and injected similarly to Group C, but ART was resumed for 3 months after vector injection. The scheduled ART interruption during lentiviral vector injection is needed for vSIV-HSV-1-tk-RRE infection, vector amplification, and mobilization in the body. The resumption of ART after vector injection in Group **d** was to inhibit uncontrolled SIV spread. ART was terminated after 3 months, and GCV was administered intravenously for two weeks to induce the killing of SIV+ cells by HSV-1-tk for animals in Groups **c** and **d**. Following GCV administration, all treatments were terminated and viral rebound was monitored. **B** Plasma viral loads (copies/ml) following SIVmac239 infection, ART, vSIV-HSV-1-tk-RRE/GCV injection, and vSIV-TRAF6(R)-RRE injection in animals of groups **a** to **d**. **C** Plasma viral loads (copies/ml) following the scheme of Group **d** with a single injection of vSIV-HSV-1-tk-RRE injection. Adapted with permission from Springer Nature Ltd. *Gene Therapy* 32, 16–24 (2025) [12]

Our selection of HSV-1-tk as the primary therapeutic gene for in vivo use is based on several key advantages: (i) Superior safety profile. In comparison with toxin-mediated cell killing, the ganciclovir (GCV)/HSV-1-tk system has been extensively studied in vitro and in vivo for over 40 years [220–224]. This approach has undergone several clinical trials, exhibiting desirable safety and efficacy for killing numerous cell types [224–226]. Notably, few side effects were observed even at high concentrations of HSV-1-tk vectors (10^10^ transduction units per injection) [225, 226]. (ii) Capability for mobilization and amplification in vivo. In the absence of GCV, HSV-1-tk vector-mediated killing will not occur. The Rev-dependent vector contains fully functional LTR, the packaging signal (ψ), and the Rev-response element (RRE), which can mobilize in the presence of HIV. (iii) CNS targeting capability via GCV. GCV has been shown to crosses the blood-brain barrier (BBB) [227], allowing CNS-specific targeting of HIV-infected cells. Targeted killing of tumor cells in the brain has been observed after HSV-tk vector intrathecal injection and systemic GCV administration in rats [228]. (iv) Capability for targeting post-mitotic cells. HIV/SIV-infected brain macrophages exhibit higher levels of DNA metabolism than normal cells. These infected cells express proliferating cellular nuclear antigen (PCNA), a marker indicative of SIV-stimulated DNA synthesis and repair activity in these non-proliferating cells [229, 230]. This HIV/SIV-stimulated DNA synthesis and repair activity can be exploited for HSV-1-tk-mediated killing. A similar approach using GCV/HSV-1-tk to kill terminally differentiated synovial lining cells has been successfully applied in a rabbit inflammatory arthritis model, effectively inhibiting inflammation and reducing joint swelling in HSV-tk–transduced knees [231]. (v) High-titer particle production. A major technique hurdle for assembling lentiviral particles carrying cytotoxic genes is the difficulty of producing high-titer viruses, even for low-toxic genes such as TRAF-6 [10, 11–12, ]. The lack of cytotoxicity of HSV-1-tk in the absence of GCV permits the production of high-titer lentiviral particles. Previous studies have demonstrated that high-titer viral particles can be produced and safely injected into animals [225, 226]. In our own studies, high-titer Rev-dependent HSV-1-tk viral particles have also been successfully assembled for animal injection [12]. We demonstrated that injection of SIV Rev-dependent HSV-1-tk particles led to inhibition of viral rebound after ART termination. A single injection of the particles resulted in a 100-1000-fold decrease in viral rebound in six animals. When three injections were given to an animal (KC50), both plasma and CNS viremia remained largely undetectable for more than two years after the termination of antiretroviral therapy (ART) (Fig. 4, Group c and d), providing the first proof-of-concept in vivo evidence that Rev-dependent lentiviral particles can be used to reduce viral reservoirs and inhibit viral rebound [12].

### HRIT-mediated inhibition of active HIV replication via defective interfering particles

Retroviral vector mobility can be achieved through co-packaging of viral genomes and homologous/non-homologous recombination. In the case of co-packaging, previous studies have shown that competing for encapsidation by lentiviral vector RNA can inhibit HIV replication [232] and reduce the number of wild-type virions to roughly one-sixth [233, 234]. In the case of recombination, even when occurring at low frequency, the recombinant viruses are expected to be defective and have lower replication advantages over the parental virus because of the large deletions in the Rev-dependent vector. Consistent with previous studies of defective particles, in a recent vaccination priming study, injection of defective interfering viral particles in monkeys significantly improved anti-SHIV (simian/human immunodeficiency virus) immune responses following SHIV challenge and led to durable suppression of SHIV viral load by 1000 fold, which resulted in significant reduction in disease [235].

Our Rev-dependent particles are among the smallest defective interfering particles designed, with a small defective genome carrying no HIV genes, but only the LTRs, the RRE, and multiple viral mRNA splicing sites. In cells, the Rev-dependent vector can compete with HIV for genome packaging and for Tat and Rev, interfering with HIV replication. Upon superinfection, HIV-producing cells can also mobilize the vector to enter more cells. Such mobilization can further inhibit HIV spread [234, 236–238].

### RHIT-induced anti-HIV immunity including neutralizing antibodies and cell-mediated anti-HIV immune responses

From our animal study, it became evident that targeting and eradicating all reservoir cells in the body is challenging [12]. Given this partial reduction of reservoir cells, inducing broad, high-quality, and persistent anti-HIV immunity becomes critically important. In natural HIV infection, induced neutralizing antibodies (NAbs) typically neutralize only their homologous viruses, with just a few elite neutralizers developing broadly neutralizing antibodies (bNAbs) [239]. While passive administration of bNAbs has demonstrated significant efficacy in reducing plasma viremia and viral reservoirs for long-term HIV remission [120–127], clinical application faces several issues. These include pre-existing viral resistance, variable antibody durability, and limited efficacy against latent HIV reservoirs. Future bNAb applications will likely require combination strategies, such as pairing with viral vector-based approaches to induce endogenous bNAbs and CD8 T cells [121, 129, 130, 130].

**Fig. 5:**
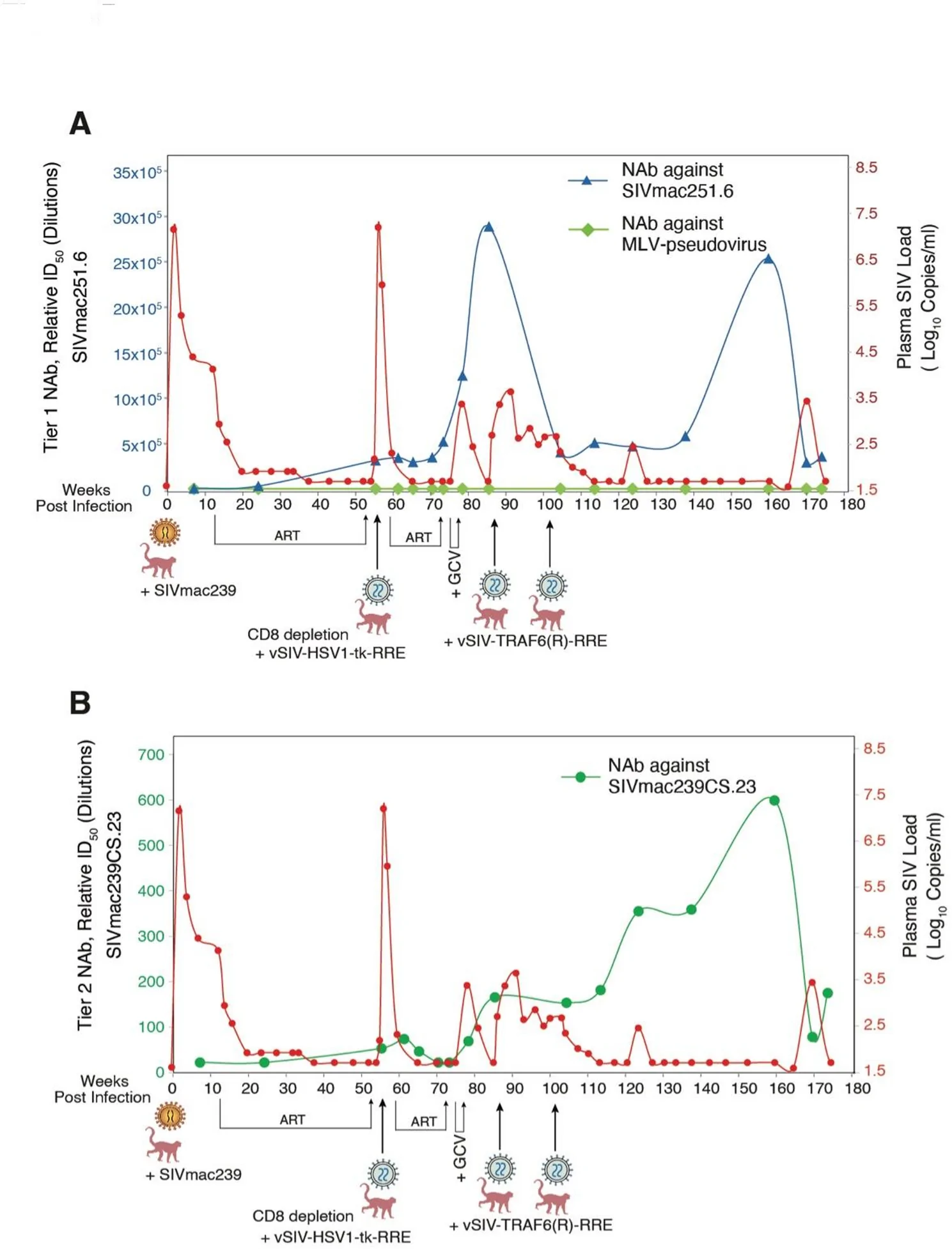
Induction of neutralizing antibodies by the Rev-dependent vector. To determine whether the injection of the SIV Rev-dependent vectors can induce the production of neutralizing antibodies (NAb), we followed KC50 infection and treatments. Serum samples from KC50 were quantified for neutralizing antibodies in pseudovirus-based antibody neutralization assays. **A** Quantification of tier 1 neutralizing antibodies using a pseudovirus-based antibody neutralization assay, which utilizes a pseudovirus carrying the envelope of the tier 1 strain SIVmac251.6 or an MLV-pseudotyped virus as a negative control. **B** Quantification of tier 2 neutralizing antibodies using a pseudovirus carrying the envelope of the tier 2 strain SIVmac239CS.23. The ID50 values represent serum dilutions at which 50% neutralization occurred. For comparison, plasma viral loads in KC50 were also plotted (red line). The background signals against MLV-pseudovirus are all < 20. Positive for neutralizing antibody activity is based on the criterion of >3x signal of the MLV control. Adapted with permission from Springer Nature Ltd. *Gene Therapy* 32, 16–24 (2025) [12]

Previous studies have revealed that the neutralization breadth of NAbs correlates with infection duration but is independent of viral inoculum properties, viral loads, or viral diversity [240]. Furthermore, studies using conditionally live-attenuated SIV mutants demonstrated that only persistent replicating virus can induce potent protective immunity against virulent viruses; only replicating attenuated SIV maintains the global T memory cell compartment profoundly skewed toward a mature effector phenotype [241]. These findings underscore the importance of persistent viral replication for inducing high-quality, long-term immunity against HIV. The Rev-dependent particle offers a unique advantage in this context: its potential to induce NAbs with expanded breadth. Superinfection of reservoir cells with Rev-dependent particles can transform them into cells that persistently release non-infectious particles, thereby stimulating anti-viral immunity. In our animal study, following partial elimination of viral reservoirs with the HSV-1-tk particle, we injected one animal (KC50) with a second Rev-dependent vector, vSIV-TRAF6(R)-RRE, which does not express a functional gene (TRAF6 ORF in the reverse orientation). Remarkably, KC50 subsequently controlled plasma and CNS viremia to undetectable levels for most of the two-year period following ART termination [12]. Mechanistic studies revealed that this persistent viremia control coincided with the induction of tier 2 neutralizing antibodies (NAbs) following Rev-dependent vector administration (Fig. 5). This result highlights the potential of the Rev vector for inducing NAbs capable of sustained viremia control.

While our animal study did not quantify anti-SIV CD8 T cells stimulated by Rev-dependent particles [12], we expect that CD8 T cells likely play an important role in suppressing viral reservoirs and preventing rebound [97, 130, 242, 243]. The long-term viremia control observed in KC50 may represent a combined effect of NAbs and cell-mediated immunity, as CD8 T cells have been shown to play a crucial role in suppressing viral reservoirs [97, 130, 242–244]. Early ART treatment during acute infection has been found to preserve HIV-specific CD8 T cell responses for long-term functionality [244]. However, in lymphoid tissues, HIV-specific CD8 T cells exhibit reduced and differentially regulated cytolytic activity [245]. An important question for future investigation is whether functional CD8 T cells can be restored when viral reservoirs are reduced in tissues by the Rev-dependent vector.

## Challenges, limitations, and future development of RHIT

### Complexity of the Rev-RRE interaction in reservoir cells

Primary HIV proviruses exhibit substantial variability in both Rev function and RRE sequence. Such variation occurs not only between isolates from different individuals but also within viral quasispecies in a single patient [246–249]. This heterogeneity presents a significant challenge for strategies that rely on Rev-dependent targeting of reservoir cells, as functional compatibility between Rev and the RRE can differ widely. Notably, recent work shows that viruses with low Rev activity can rapidly acquire compensatory mutations in the RRE that restore or even enhance Rev-RRE function and viral replication [250]. For instance, a spontaneous RRE mutation (C7214T) within the primary Rev-binding region is sufficient to rescue a low-activity 8-G Rev variant, enabling high-level viral replication [250]. These findings highlight the functional plasticity of the Rev-RRE axis and suggest a potential path forward: engineering optimized RRE variants capable of interacting efficiently with diverse Rev variants, thereby improving the breadth and efficacy of Rev-dependent vectors for targeting heterogeneous reservoir populations. In addition, enhanced Rev-RRE interactions that increase nuclear export of unspliced RNA may also improve the availability of packageable vector transcripts and thereby enhance selective packaging of the Rev-dependent vector genome in reservoir cells.

### The leakiness of the Rev-RRE regulated gene expression

A significant challenge to the express of cytotoxic genes is “leakiness,” defined as low-level transgene expression in the absence of functional Rev. This background activity can arise from incomplete nuclear retention of unspliced transcripts, cryptic splicing that bypasses Rev dependence, or basal promoter activity that drives low-level expression even in uninfected cells. Several strategies can mitigate this issue and tighten restriction to HIV⁺ cells. At the RNA level, Rev dependence can be reinforced by incorporating strong nuclear retention features (e.g., suboptimal splice donor/acceptor sites or retention motifs), thereby ensuring that transcript export remains strictly Rev-dependent. In parallel, elimination of cryptic splice sites and unintended open reading frames can reduce opportunities for Rev-independent expression. At the protein level, the inclusion of degron or destabilization domains can promote rapid degradation of any low-level toxin produced in the absence of robust Rev activity. In our animal study, we employed HSV-1-tk as the therapeutic gene [12]. A key advantage of this system is that HSV-1-tk is non-cytotoxic in the absence of ganciclovir (GCV), allowing temporal control of cell killing and providing an additional safety layer.

### Delivery challenges

Efficient delivery of Rev-dependent vectors to HIV reservoir cells remains a major challenge, particularly in anatomically and immunologically protected sites such as lymphoid tissues and the central nervous system, as well as within deeply latent cell populations. In particular, some latent reservoirs may harbor proviruses integrated into genomic sites that do not support expression of viral proteins, including Tat and Rev [67, 155], potentially limiting vector activation. Possible approaches to address these hurdles include engineering vector tropism to enhance delivery specificity, improving particle stability and biodistribution, and developing ex vivo delivery strategies using engineered autologous CD4⁺ T cells. In addition, the low frequency of reservoir cells and the absence of Tat and Rev expression in deeply latent reservoirs may reduce overall efficacy. To overcome these constraints, combination strategies may be required, integrating Rev-dependent targeting with latency-reversing agents, immune-based interventions, or gene-editing technologies to achieve greater reservoir reduction. Nevertheless, such stimulation may not always be necessary, as the Rev-dependent vector may persist as a provirus in latent cells and be reactivated upon autonomous proviral activation. Furthermore, to minimize silencing of the Rev-dependent vector caused by integration into transcriptionally inactive chromatin, the vector integrase (IN) could be engineered to favor integration into more transcriptionally active regions. Potential approaches include generating IN mutants with enhanced interaction with LEDGF/p75 to promote targeting of H3K36me3-rich active chromatin, or modifying IN-host factor interactions to bias integration toward transcription factories or nuclear speckle-associated domains (SPADs) [251, 252]. Such strategies may improve sustained vector expression and responsiveness upon reservoir reactivation.

### Improving safety using non-integrating Rev-dependent vector

Although lentiviral vectors are being used in clinical trials for a range of human diseases, insertional mutagenesis associated with lentiviral integration remains a potential safety concern. To mitigate this risk, a non-integrating Rev-dependent vector can be employed. An additional advantage of this approach is that it may eliminate the need for complete interruption of antiretroviral therapy during vector administration, as integrase inhibitors can be maintained, thereby reducing risks associated with ART interruption. Previous studies have shown that non-integrating Rev-dependent vectors carrying the integrase D116N mutation can efficiently infect cells and express reporter or therapeutic genes selectively in HIV-infected cells [217, 253–257]. Moreover, these vectors retain fully functional LTRs, the packaging signal, and RRE, resembling a previously described conditionally replicating lentiviral vector capable of mobilization in the presence of HIV [238].

## Conclusions and perspective

For more than a decade, HIV cure research has been dominated by the “shock and kill” paradigm, predicated on the assumption that a drug-mediated transient activation of the immune system would lead to the eradication of HIV latent reservoirs. More recently, immune-based therapies, particularly the passive administration of bNAbs, have emerged as viable approaches for achieving long-term viral remission in the absence of a sterilizing cure. The existence of elite controllers demonstrates that long-term suppression of HIV is biologically achievable without a sterilizing cure. However, elite control remains rare, and the mechanisms underlying this phenotype are incompletely understood [258–260]. Growing evidence suggests that viral reservoir size, composition, and anatomical distribution, particularly within tissues such as lymph nodes, gut-associated lymphoid tissue, and other immune-privileged sites, play a central role in determining whether immune control can be sustained [40, 261]. A deeper understanding of these tissue reservoirs may therefore be key to designing immunotherapies capable of extending elite-controller-like control to the broader population of people living with HIV.

In this context, virus-informed therapeutic strategies that directly exploit features of HIV persistence warrant renewed attention. The Rev-dependent vector exemplifies such an approach by aligning therapeutic activity with active viral gene expression, thereby selectively targeting infected reservoir cells while sparing uninfected bystanders. This specificity distinguishes RHIT from broad immune activation strategies, which often carry substantial risks of off-target effects, immune exhaustion, and systemic toxicity. By minimizing collateral damage, RHIT offers a potentially safer means of reshaping host-virus dynamics in favor of long-term immune control.

Importantly, RHIT is not envisioned as a standalone cure, but rather as a component of combination cure strategies. It could be deployed alongside suppressive antiretroviral therapy, immune-based interventions such as broadly neutralizing antibodies, or latency-modulating agents that transiently increase viral transcription within reservoirs. Such combinations may allow therapeutic pressure to be focused precisely where viral persistence is maintained, particularly within tissues that are poorly accessed by conventional therapeutics.

Translating RHIT into a clinically viable intervention will require further optimization. Vector optimization remains a priority, including the development of safer, non-integrating vectors capable of mobilization in provirus-harboring reservoir cells [11, 256, 262]. Equally important is the establishment of practical delivery strategies. Ex vivo modification of autologous hematopoietic stem cells or CD4⁺ T cells offers a controlled and potentially durable route, while in vivo delivery using manufactured lentiviral particles could enable broader and more scalable access. Ultimately, progress toward a functional HIV cure may depend less on eliminating every last provirus than on redefining the equilibrium between host immunity and viral persistence. Therapeutic strategies that are informed by the biology of reservoirs, particularly within tissues, may allow immune control to be engineered rather than discovered by chance. In this emerging landscape, RHIT represents one example of how virus-aligned design principles could help bridge the gap between elite control and population-wide remission [12].

## Data Availability

No datasets were generated or analysed during the current study.
